# Effects of different pickling methods on physicochemical properties and flavor profiles of Tongling white ginger: Dry‐salting, brine‐pickling, and inoculation‐pickling

**DOI:** 10.1002/fsn3.3942

**Published:** 2024-01-09

**Authors:** Kaili Zong, Feixiang Jin, Daquan Wang, Hongchao Hu, Haipeng Cui, Jianting Yang

**Affiliations:** ^1^ Food Engineering College Anhui Science and Technology University Chuzhou China

**Keywords:** brine pickling, dry salting, flavor, inoculation pickling, Tongling white ginger

## Abstract

Tongling white ginger is a Chinese fermented vegetable with unique flavors. However, little is known about its physicochemical properties, flavor characteristics, and sensory evaluation. The study examined the physicochemical (pH, titratable acidity [TA], nitrite, soluble protein, and color) and flavor characteristics (organic acids, free amino acids, and volatiles) of white ginger during fermentation. The results showed that the pH value and soluble protein in the dry‐salted, brine‐pickled, and inoculation‐pickled decreased significantly while the TA value increased significantly, inoculation‐pickled can effectively reduce the content of nitrite. After fermentation, inoculation‐pickled produced the highest content of organic acids, while dry‐salted produced the highest total amount of free amino acids. A total of 70, 68, 70, and 69 volatile compounds were identified in fresh, dry‐salted, brine‐pickled, and inoculation‐pickled white ginger. The total contents of terpenoids of Tongling white ginger by three fermentation methods decreased; the total contents of alcohols and aldehydes were the highest in brine‐pickled, and esters and ketones were more abundant in inoculation‐pickled. The results showed that inoculation‐pickled could shorten the fermentation time of Tongling white ginger, produce a unique flavor, and have the highest sensory score.

## Graphical abstract

Our study revealed the physicochemical properties and flavor characteristics of dry‐salted, brine‐pickled, and inoculation‐pickled white ginger. The results showed that inoculation pickling could shorten the fermentation time, reduce the content of nitrite, and increase the flavor richness of Tongling white ginger.

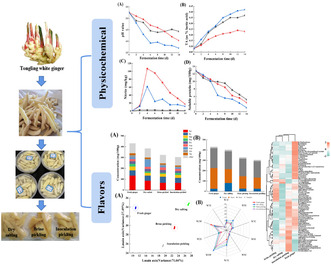

## 1 INTRODUCTION

The ginger plant (*Zingiber officinale* Roscoe) belongs to the Zingiberaceae family of plants and is rich in bioactive ingredients with antioxidant, anti‐inflammatory, and anti‐cancer effects (Jafarzadeh & Nemati, 2018, Zhang, Thakur, et al., 2017; Zhang, Zhang, et al., 2017). Tongling white ginger is a geographically indicated product from Anhui, China. After thousands of years of cultivation, white ginger has long become well known for its large size, thin skin, abundant juice, low residue, and rich flavors (Zhang, Thakur, et al., 2017; Zhang, Zhang, et al., 2017). Although ginger is favored for its unique flavor and texture, it is difficult to store due to its high moisture content. To meet the demand for consumption during the non‐harvesting period, white ginger is often pickled for storage. People in Sichuan Province, China, have traditionally consumed ginger paocai, a traditional pickled vegetable (Xiong et al., 2012). In China, the production of paocai often involves a long fermentation process using brine (Liu et al., 2015). Pickled ginger not only improves the flavor of ginger but also its nutritional value.

In the fermentation process, raw materials and environmental factors can influence the flavor of pickles (Wu et al., 2015). The flavor is one of the characteristics that better reflect consumers' initial impressions of quality indicators. Currently, there are many studies on ginger flavor. For example, Yu et al. (2007) optimized headspace solid‐phase microextraction (HS‐SPME) conditions for ginger volatiles. Pang et al. (2017) identified the volatiles and aroma‐active components in ginger. Ren et al. (2021) and Yu et al. (2022) investigated how different drying techniques affected the volatile substances in ginger. However, studies on the flavor of pickled Tongling white ginger are very rare.

Two widely used processing methods are dry salting and brine pickling. Typically, dry salted vegetables are fermented with a relatively low moisture content after being partially dehydrated and mixed with salt. Greek dry‐salted olives are one example of a dry‐salted vegetable (Değirmencioğlu et al., 2011), Chinese dry‐salted radish (Li et al., 2020), and Japanese takuan‐zuke (Kumakura et al., 2017). Usually, brine‐pickled vegetables are made by fermenting fresh vegetables in brine, either naturally or by bacteria. Examples of brine‐pickled vegetables include Chinese pickled vegetables (Rao et al., 2020), Korean kimchi (Lee et al., 2019), and European sauerkraut (Satora et al., 2021).

Vegetables may exhibit differences in sensory, nutritional, and flavor properties after dry‐salting, brine‐pickling, or inoculation‐pickling. However, the impact of these processes on the quality and volatile compound profiles of salted Tongling white ginger remains uncertain. Dry‐salted, brine‐pickled, and inoculation‐pickled white ginger were prepared and fermented for 14 days for this study. Analyses were conducted to evaluate the physicochemical properties, nonvolatile flavor compounds, volatile flavor compounds, and sensory evaluation.

## 2 MATERIALS AND METHODS

### 2.1 Materials

Tongling white ginger was collected 6 months after planting and purchased from the white ginger plantation in Tongling City. The salt used in the study was food‐grade from Shandong Zhenghao Salt Technology Co., Ltd. The *Lactobacillus plantarum* powder used in the study had an activity of 1.0 × 10^10^ cfu/g. It was purchased from Xi'an Jushengyuan Biological Technology Co., Ltd. Other chemicals used were analytical‐grade.

### 2.2 Preparation of dry‐salted, brine‐pickled, and inoculation‐pickled Tongling white ginger

First, wash the white ginger and cut it into pieces that are 8 mm thick, and then prepare by dry‐salting, brine‐pickling, and inoculation‐pickling. For the dry‐salting process, approximately 80% moisture should be retained in the white ginger after air drying. Mix the dehydrated white ginger with dry salt in a 5:1 ratio and store it in a sealed container. For the brine‐pickling process, mix the white ginger with 8% salt water in a 1:1 ratio. For the inoculation‐pickling process, add *Lactobacillus plantarum* powder to the white ginger. The amount of powder added should be 2.2% of the mass of the ginger. Store them at a constant temperature for 14 days. Every 2 days, randomly sample the white ginger from each salting method. The sample was aseptically packaged and stored at −80°C for later quality analysis. Repeat the preparation experiments for each of the three salting methods three times in parallel.

### 2.3 pH, titratable acidity (TA), and nitrite

The pH, TA, and nitrite contents of samples were measured by homogenizing them in a blender. A P901 pH meter was used to measure pH (Shanghai, China). TA was determined using the titration method described in AOAC 942.15. Nitrite content was determined through spectrophotometry, as outlined in GB 5009.33–2016.

### 2.4 Color

The color parameters (lightness L*, chroma a*, and chroma b*) of the upper surface of the Tongling white ginger were measured using a colorimeter on the CIEL*ab scale (Hunterlab, Miniscan EZ, E.U.A), with ten replicates for the samples. The lightness (L*, ranging from 0 for black to 100 for white), redness (+a*), greenness (−a*), yellowness (+b*), and blueness (−b*) values were recorded. The following equation was used to calculate the total color difference (ΔE):

$$∆E=\sqrt{\left[{\left(L^{*}-L_{0}^{*}\right)}^{2}+{\left(a^{*}-{\;a}_{0}^{*}\right)}^{2}+{\left(b^{*}-b_{0}^{*}\right)}^{2}\right]}$$

The color parameters of the white standard plate were *L*_0_* = 99.50, *a*_0_* = −0.06, and *b*_0_* = −0.19.

### 2.5 Organic acids

The white ginger was analyzed for eight organic acids, including oxalic acid, formic acid, malic acid, lactic acid, acetic acid, maleic acid, citric acid, succinic acid, and propanoic acid, using HPLC with UV detection. The fermented 14‐day white ginger and fresh white ginger were tested and analyzed. The separation process was carried out on an Agilent column (5 μm, 250 mm × 4.6 mm) at a temperature of 30°C. The mobile phase consisted of a mixture of acetonitrile and water (with a potassium dihydrogen phosphate concentration of 0.05 mol/L and phosphoric acid, pH 2.68) in a ratio of 0.5:99.5. The UV detector was adjusted at a wavelength of 210 nm, and the mobile phase flow rate was kept at 0.5 mL/min.

### 2.6 Soluble protein and free amino acids

The soluble protein content was performed using the Coomassie brilliant blue G250 method (Ye et al., 2020). As a standard, bovine serum albumin was measured using a UV‐spectrophotometer (T9CS, Persee, China). The fermented 14‐day white ginger and fresh white ginger were tested and analyzed. All measurements were performed in triplicate.

Two gram of samples (fermented 14‐day white ginger and fresh white ginger) were taken in a 50‐mL volumetric bottle, dissolved fully, water was added to the scale, mixed well, and left for 24 h. Next, 20 mL of the supernatant and 20 mL of a 5% sulfosalicylic acid solution were added to a 10‐mL centrifuge tube. A 6000 *g* centrifuge was used for 10 minutes to centrifuge the mixture, and 20 mL of the supernatant was evaporated to dryness in a rotary evaporator. 1 mL of sodium citrate buffer solution was used to dissolve dried supernatant and the sample was filtered using a 0.45‐μm membrane. The Biochrom 30+ amino acid analyzer was used for the analysis, with a flush flow rate of 20 mL/h and a reaction flow rate of 10 mL/h. The separation column used was a Na‐type cationic resin chromatography column with a length and diameter of 200 mm × 4.6 mm. The UV detection wavelengths were set to 570 nm and 440 nm, and the column temperature was programmed to 55–65–77°C. The reaction tank temperature was set to 138°C, and the sample size used was 50 μL. The amino acid analysis was performed by separating the amino acids using the column and reacting them with ninhydrin. The resulting product was then detected using a spectrophotometer to determine the concentration of the amino acid.

### 2.7 Electronic nose

The PEN 3 electronic nose (AIRSENSE, Germany) is comprised of a sampling apparatus, a detector unit housing ten distinct metal oxide sensors, and pattern recognition software (Win‐Muster) for the purpose of data logging and analysis. The sensor was capable of detecting cross‐sensitive olfactory information (Zhu et al., 2020). Each sensor exhibits specific response characteristics, as outlined below: W1C (aromatic compounds), W5S (nitrogen oxide), W3C (ammonia and aromatic compounds), W6S (hydrogen compounds), W5C (short‐chain alkane aromatic compounds), W1S (methyl group), W1W (inorganic sulfides), W2S (alcohols, aldehydes, and ketones), W2W (aromatic compounds and organic sulfides), and W3S (long‐chain alkanes).

1 g of sample (fermented 14‐day white ginger and fresh white ginger) was placed in a sample bottle. After 30 min, the electronic nose probe was inserted into the bottle to capture the top air and determine the volatile substances present. The electronic nose was set to the following parameters: a test time of 60 s, a cleaning time of 60 s, internal flow of 600 mL/min, and a sampling flow of 600 mL/min.

### 2.8 Volatile fraction

Liquid nitrogen was used to grind the samples (fermented 14‐day white ginger and fresh white ginger). To prevent enzyme reactions, the powder (5 g) was immediately transferred into a NaCl‐saturated solution in a 20‐mL headspace vial (Agilent, USA). The vials were heated to 60°C for 5 min. A 120‐m fiber was then exposed for 15 min at 100°C to the sample's headspace.

A splitless mode of 250°C for 5 min was used to desorb volatile organic compounds (VOCs) from the fiber coating after sampling by the GC apparatus (Model 8890; Agilent). With an Agilent Model 8890 gas chromatograph (GC) and an Agilent Model 7000D mass spectrometer (Agilent), VOCs were identified and quantified. Using helium at 1.2 mL/min as the carrier gas, a 280°C detector was used in conjunction with a 250°C injector. Temperatures in the oven were increased over 5 min from 40°C to 100°C/min, then to 180°C at 7°C/min, and then to 280°C at 25°C min and held for 5 min. A 70 eV electron impact ionization (EI) mode was used to record the mass spectrum. Temperatures were set at 150°C, 230°C, and 280°C for the quadruple mass detector, ion source, and transmission line, respectively.

### 2.9 Sensory evaluation

White ginger sensory evaluation was conducted following Chen, Luo, Peng, et al. (2019); Chen, Luo, Niu, et al. (2019) and Ye et al. (2020) with modifications according to the product features. White ginger and brine in a ratio of 1:1 were presented with samples in a random order to ten assessors, including six females and four males with an average age of 30 years. The panelists consisted mainly of students and staff from the School of Food Engineering who agreed to participate in the sensory evaluation. Before the formal evaluation, training was conducted three times to obtain objective results. Color, odor, crispiness, taste, and overall acceptability were evaluated using a 10‐point scale ranging from 1 (dislike extremely) to 10 (like extremely).

### 2.10 Data analysis

All data were analyzed for significance using SPSS 25.0 statistical software. Principal component analysis was done using Winmuster software. The graphs were generated using Origin (2023).

## 3 RSEULTS AND DISCUSSION

### 3.1 pH and TA values

**FIGURE 1:**
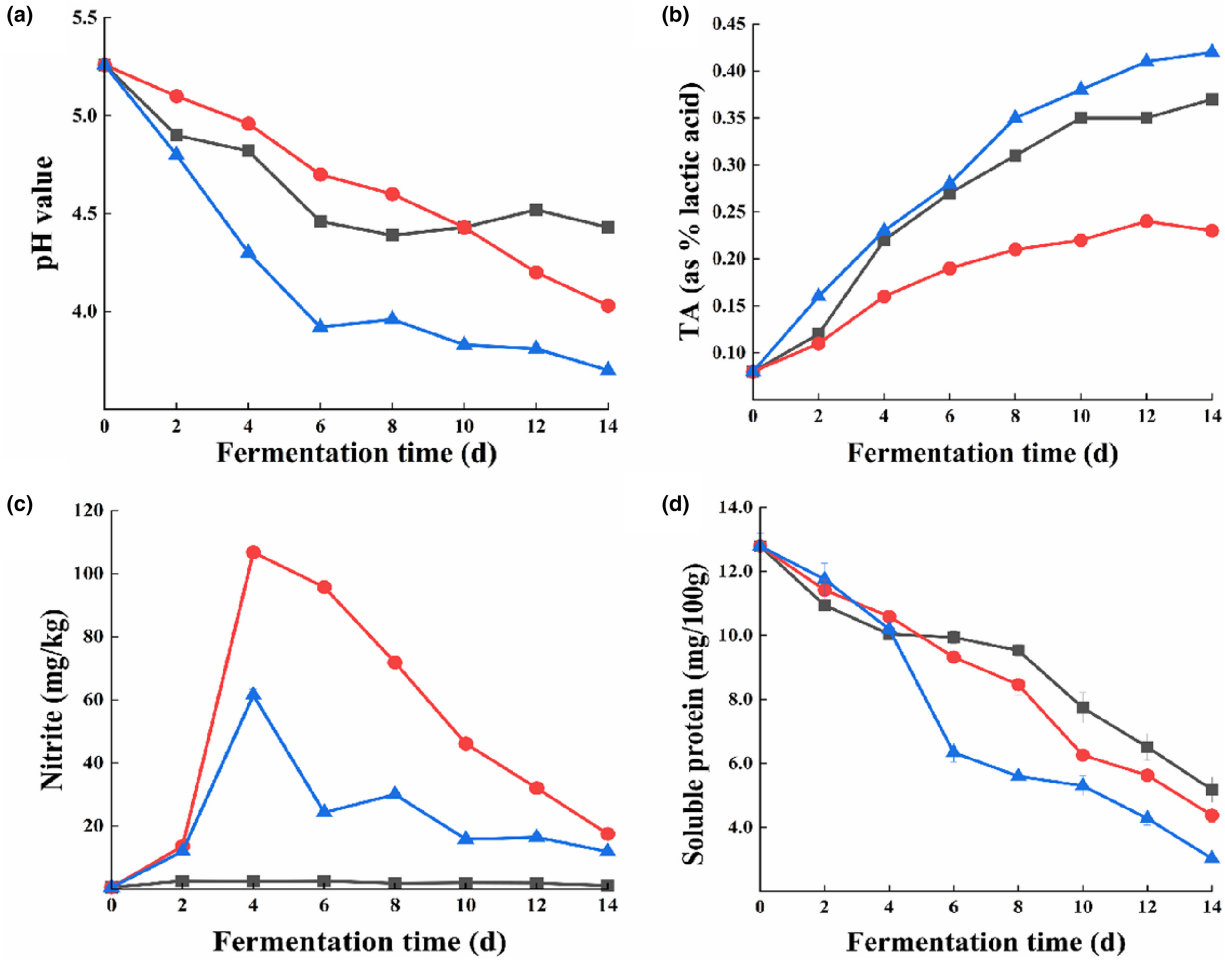
Changes in physicochemical properties (a: pH value; b: TA; c: nitrite; d: soluble protein) of Tongling white ginger during fermentation for 14 days. The symbols and their representative groups are as follows: ■ Dry salting; ● Brine pickling; ▲ Inoculation pickling.

The pH and TA values are the main indicators of microbial growth and microbial metabolite accumulation (Wu et al., 2015). According to Figure 1a,b, fresh white ginger has a pH of 5.26. During the fermentation process, the pH of the dry‐salted white ginger decreased to 4.46 and stabilized until the end of the fermentation process, with TA increasing from 0.08% to 0.44%. The pH value remained in the range of 4.39–4.56 from day 8 to day 12, which agrees with the findings provided by other researchers (Chi et al., 2020). Brine‐pickled white ginger's pH gradually dropped during fermentation until it reached 4.03 at the end, and TA stabilized after reaching 0.21% at 8 days. In the case of inoculation‐pickled white ginger, the pH value dropped sharply in the pre‐fermentation stage, reaching 3.92 after 6 days of fermentation with a TA of 0.28%. With the extension of the fermentation time, the pH remained basically unchanged. Similar results were reported by Xiong et al. (2016), who found that after 96 hours of fermentation, the pH of Chinese brine‐pickled cabbages fell sharply from 6.4 to 3.5 and then almost remained stable. This could occur as a result of the fermentation process limiting the growth of lactic acid bacteria (Badwaik et al., 2014). Inoculation‐pickling promotes the generation of lactic acid in the early stage of fermentation, resulting in a sharp decline in pH. Therefore, compared to dry‐salting and brine‐pickling, inoculation‐pickling is more beneficial for accelerating vegetable fermentation and shortening the maturity period.

### 3.2 Nitrite content

Pickle fermentation can result in nitrite formation and accumulation, which are human health hazards (Ding et al., 2018). Figure 1c depicts the amount of nitrite content in fermentation. The nitrite content of fresh Tongling white ginger was 0.53 mg/kg; after 4 days, brine‐pickling, and inoculation‐pickling reached the highest values of 106.81 mg/kg and 61.51 mg/kg, respectively. It may be caused by non‐acidic microorganisms reducing nitrate on the surface of fresh white ginger (Wu et al., 2015). From the Figures, the nitrite content in inoculation‐pickling was significantly lower than that in brine‐pickling, indicating that inoculation‐pickling improves the safety of the product and effectively produces healthy, safe white ginger products. The nitrite content gradually decreased with an extended period of fermentation. The amount of nitrite in the dry‐salting during the entire fermentation process was no more than 2.6 mg/kg. At 12 days of fermentation, the nitrite contents of dry‐salting, brine‐pickling, and inoculation‐pickling were 1.91, 32.4, and 16.42 mg/kg, respectively. On day 14 of fermentation, the nitrite contents of dry‐salting, brine‐pickling, and inoculation‐pickling were 1.15, 17.53, and 11.9 mg/kg, respectively. However, depending on China's National Health Regulation for nitrite (20 mg/kg), this study's experiment with fermented white ginger was found safe for ingestion (Ye et al., 2020).

### 3.3 Color

Table 1 presents the observed alterations in color for Tongling white ginger subjected to three different preservation methods, namely dry‐salting, brine‐pickling, and inoculation‐pickling, over a span of 14 days. The color parameters L*, a*, and b* have beginning values of 68.38, 0.50, and 23.49, respectively. Notably, the L* value of dry‐salted white ginger exhibited a substantial decrease throughout the fermentation process, the a* value increased significantly, and the b* value fluctuated less, indicating that the color of dry‐salted white ginger became darker. The L* value and b* value of brine‐pickled white ginger changed little, with a slight decrease, while the a* value increased significantly, indicating that the redness of brine‐pickled white ginger increased. The L* value of white ginger inoculation‐pickled decreased significantly, the a* value increased significantly, and the b* value decreased slightly. The observation suggests that the color of white ginger inoculation‐pickled exhibited a deepening and reddening effect. The total color difference (ΔE) serves as a significant quality parameter primarily employed for assessing color changes in food items (Yang, Fan, et al., 2022; Yang, Wang, et al., 2022). In this study, the ΔE* of dry‐salted, brine‐pickled, and inoculation‐pickled white ginger changed significantly, revealing that the brightness of the fermented white ginger was reduced compared to the fresh white ginger and that the color difference was more obvious. However, it was still bright ginger‐yellow. The alteration in color observed in Tongling white ginger during fermentation ascribed to the interplay between microbial activity and chemical reactions; the chemical reactions involved primarily encompass non‐enzymatic browning, enzymatic browning, and the Maillard reaction (Chi et al., 2020). White ginger contains higher moisture, and high moisture conditions are prone to Maillard reactions, which promote the browning of fermented vegetables (Yunwei et al., 2020).

**TABLE 1:** Color parameter changes during the fermentation process.

Fermentation time (day)								
	0	2	4	6	8	10	12	14
Dry salting								
L	68.38 ± 0.51^a^	65.59 ± 0.73^ab^	66.17 ± 0.42^a^	65.75 ± 0.68^a^	64.58 ± 0.26^b^	63.24 ± 0.13^c^	61.19 ± 0.27^d^	58.93 ± 0.63^e^
a	0.50 ± 0.02^f^	0.77 ± 0.06^e^	0.87 ± 0.02^e^	1.12 ± 0.14^d^	1.19 ± 0.07^d^	1.40 ± 0.05^c^	1.58 ± 0.04^b^	2.09 ± 0.14^a^
b	23.49 ± 0.36^ab^	23.53 ± 0.10^ab^	22.84 ± 0.68^bc^	23.93 ± 0.31^a^	20.69 ± 0.23^d^	22.35 ± 0.40^c^	20.81 ± 0.11^d^	19.63 ± 0.43^e^
ΔE	39.11 ± 0.24^f^	47.64 ± 0.47^c^	46.55 ± 0.39^d^	47.93 ± 0.22^bc^	45.67 ± 0.03^e^	48.22 ± 0.27^bc^	48.42 ± 0.17^b^	49.26 ± 0.39^a^
Brine pickling								
L	68.38 ± 0.51^a^	65.70 ± 0.11^e^	66.40 ± 0.42^de^	67.13 ± 0.67^bcd^	67.44 ± 0.54^bc^	66.68 ± 0.29^cd^	67.78 ± 0.43^ab^	67.72 ± 0.18^ab^
a	0.50 ± 0.02^f^	0.54 ± 0.05^f^	1.12 ± 0.07^e^	2.34 ± 0.15^d^	2.51 ± 0.15^d^	3.19 ± 0.07^c^	3.31 ± 0.39^b^	3.72 ± 0.08^a^
b	23.49 ± 0.36^a^	22.30 ± 0.85^bc^	22.43 ± 0.50^b^	21.53 ± 0.21^cd^	22.51 ± 0.48^b^	21.26 ± 0.17^d^	21.45 ± 0.35 cd	21.38 ± 0.20^d^
ΔE	39.11 ± 0.24^bc^	40.60 ± 0.39^a^	40.11 ± 0.09^a^	39.06 ± 0.47^bcd^	39.37 ± 0.19^b^	39.34 ± 0.17^b^	38.56 ± 0.24^d^	38.60 ± 0.04^cd^
Inoculation pickling								
L	68.38 ± 0.51^a^	66.71 ± 0.15^b^	64.41 ± 0.49^cd^	63.84 ± 0.81^d^	63.75 ± 0.51^d^	64.84 ± 0.20^c^	63.55 ± 0.23^d^	62.58 ± 0.01^e^
a	0.50 ± 0.02^e^	0.77 ± 0.07^e^	1.13 ± 0.08^d^	2.06 ± 0.10^c^	2.43 ± 0.08^b^	2.77 ± 0.43^a^	2.25 ± 0.08^bc^	2.98 ± 0.05^a^
b	23.49 ± 0.36^a^	22.63 ± 0.40^b^	21.31 ± 0.23^de^	21.79 ± 0.16^cd^	22.11 ± 0.25^bc^	20.53 ± 0.77^e^	21.52 ± 0.37^cd^	21.26 ± 0.35^de^
ΔE	39.11 ± 0.24^f^	39.96 ± 0.11^e^	41.17 ± 0.50^cd^	41.94 ± 0.76^bc^	42.21 ± 0.29^ab^	40.49 ± 0.29^de^	42.06 ± 0.37^ab^	42.81 ± 0.18^a^

^a–f^ Values followed by different letters in the same column were significantly (*p* < .05) different, where “a” represents the maximum value.

### 3.4 Organic acids

Organic acids contribute significantly to the sour flavor of pickled vegetables. In this study, we found nine organic acids in Tongling white ginger, namely oxalic, lactic, formic, malic, lactic, acetic, maleic, citric, succinic, and propanoic acids.

Table 2 shows that fresh Tongling white ginger had the highest lactic acid content, reaching 272.32 mg/100 g. However, oxalic acid and malic acid were not detected in fresh ginger. Dry‐salting resulted in a significant reduction in formic acid, lactic acid, acetic acid, succinic acid, and propanoic acid. Malic acid was detected at a concentration of 18.89 mg/100 g during the dry‐salting process, which aligns with previous research indicating that malic acid is commonly found in dry‐salted vegetables (Tang et al., 2022). Chen, Luo, Peng, et al. (2019); Chen, Luo, Niu, et al. (2019) have reported that the roots and stems of vegetables are where these organic acids are mostly found, and their formation in a dry‐salting environment is facilitated by microbial metabolism and endogenous enzymes (Lin et al., 2021). During the brine‐pickling process, only four organic acids were detected: formic acid, malic acid, lactic acid, and acetic acid. Compared to fresh ginger, the content of these four organic acids decreased significantly. Inoculation‐pickling detected oxalic acid, and its content was 24.45 mg/100 g. There was a high concentration of lactic acid in brine‐pickling and inoculation‐pickling, which may have been caused by the activity of lactic acid bacteria. Research confirms that in brine‐pickled vegetables, the most abundant organic acid is lactic acid (Satora et al., 2021) and Sichuan pickles (Rao et al., 2020). After three different fermentation methods, the content of most organic acids decreased significantly, and this phenomenon was also seen in Yang, Wang, et al. (2022), Yang, Fan, et al. (2022) and Shang et al. (2022). This may be because organic acids, such as lactic acid, act as a carbon source for microbial growth, resulting in its reduction. It may also be due to differences in raw materials and microbial composition. The total amount of organic acids in different fermentation methods was as follows: inoculation‐pickling > brine‐pickling > dry‐salting. Adding *Lactobacillus plantarum* powder can help form non‐volatile characteristics.

**TABLE 2:** Concentration of organic acids during the fermentation process.

Organic acids (μg/g)	Sample			
	Fresh ginger	Dry salting	Brine pickling	Inoculation pickling
Oxalic acid	–	–	–	24.45 ± 2.32^a^
Formic acid	61.00 ± 2.05^a^	31.82 ± 1.26^c^	44.59 ± 0.66^b^	45.80 ± 3.88^b^
Malic acid	–	18.89 ± 0.66^a^	3.86 ± 0.14^b^	–
Lactic acid	272.32 ± 9.70^a^	8.32 ± 1.19^d^	52.82 ± 3.87^c^	72.77 ± 4.38^b^
Acetic acid	44.24 ± 0.39^a^	7.04 ± 0.22^c^	12.07 ± 0.51^b^	2.80 ± 0.07^d^
Maleic acid	0.11 ± 0.00^a^	–	–	0.07 ± 0.00^a^
Citric acid	4.02 ± 0.06^a^	–	–	–
Succinic acid	4.26 ± 0.19^a^	3.06 ± 0.01^b^	–	–
Propanoic acid	36.17 ± 0.19^a^	21.08 ± 0.52^b^	–	14.82 ± 0.81^c^

^a–d^ Values followed by different letters in the same column were significantly (*p* < .05) different, where “a” represents the maximum value. (“–”, could not be detected.).

### 3.5 Soluble protein and free amino acid content

The primary element responsible for the exquisite flavor of pickled vegetables is free amino acids. Lactic acid bacteria contribute to the degradation of soluble proteins during fermentation, resulting in the formation of diverse free amino acids (Wu et al., 2015). The findings of this investigation reveal a notable reduction in soluble protein levels (Figure 1d).

**FIGURE 2:**
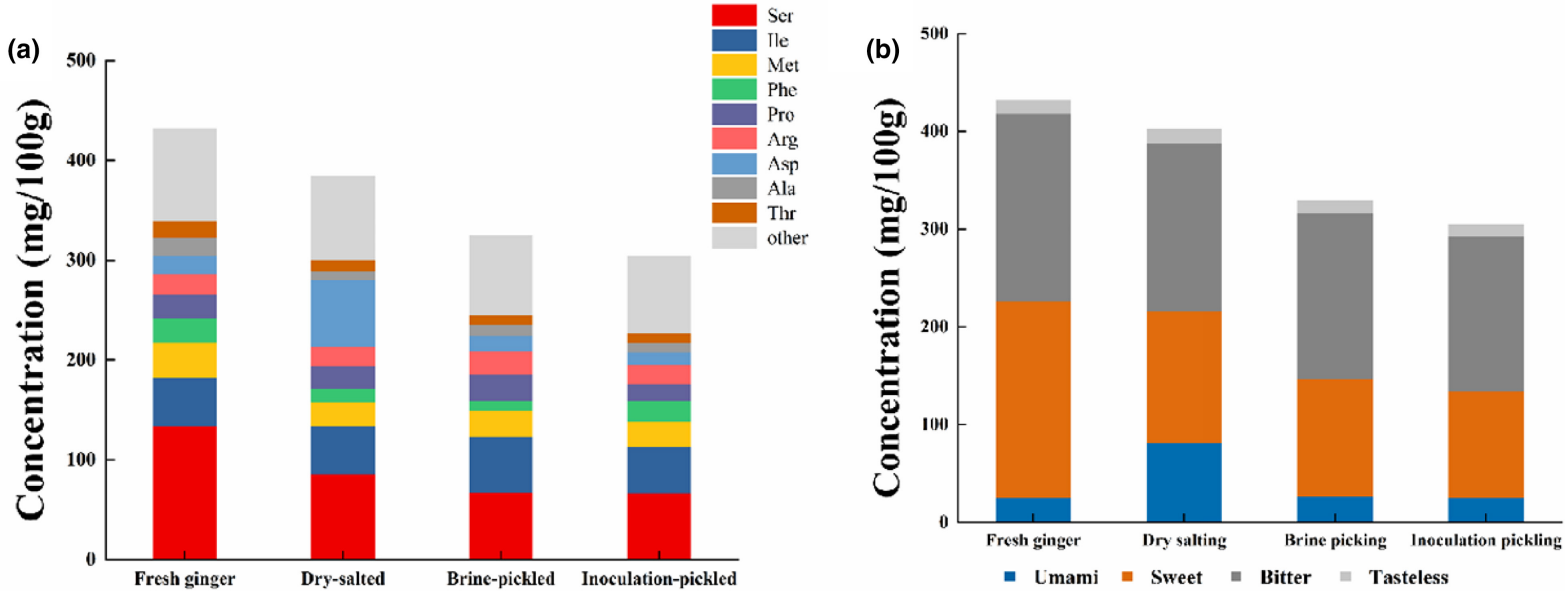
Free amino acid content of different fermented Tongling white ginger.

Figure 2a,b demonstrates that dry‐salted, brine‐pickled, and inoculation‐pickled white ginger contained 17 different types of free amino acids. Fresh ginger, on the other hand, was found to contain mainly Ser (133.40 mg/100 g), Ile (48.60 mg/100 g), and Met (35.30 mg/100 g). During fermentation of Tongling white ginger, most free amino acids' concentrations dropped, except for Asp, which significantly increased during dry‐salting. Following fermentation, the total amount of free amino acids was greater than with brine‐pickling and inoculation‐pickling. The overall quantity of free amino acids significantly decreased following brine‐pickling and inoculation‐pickling, aligning with previous observations in the fermentation of other vegetables (Jung et al., 2018; Rao et al., 2020). There is a possibility that this is related to the organic ingredients that are released into the brine during the fermentation of the white ginger raw material. As lactic acid bacteria converted the reduced sugar exudates into organic acids, there was no excess sugar to produce amino acids.

According to An et al. (2021), the four taste categories to classify free amino acids were umami (Glu and Asp), sweetness (Thr, Ser, Gly, Ala, and Pro), bitterness (Tyr, Leu, Ile, Val, Phe, Lys, His, Arg, and Met), and tastelessness (Cys). As shown in Figure 2b, sweet amino acids were more abundant in fresh ginger, after which came bitter amino acids. Dry‐salting, brine‐pickling, and inoculation‐pickling of white ginger led to an increase in umami amino acids, while the concentrations of bitter and sweet amino acids significantly decreased. However, compared to sweet and umami amino acids, bitter amino acids continued to have the highest concentration. Even though free amino acids that cause bitterness are present, the high salt and acidity in kimchi suppress the perception of bitterness. In contrast, bitter amino acids were of great significance to the formation of pickled vegetable flavor (Chen, Luo, Niu, et al., 2019; Chen, Luo, Peng, et al., 2019).

### 3.6 Electronic nose analysis

**FIGURE 3:**
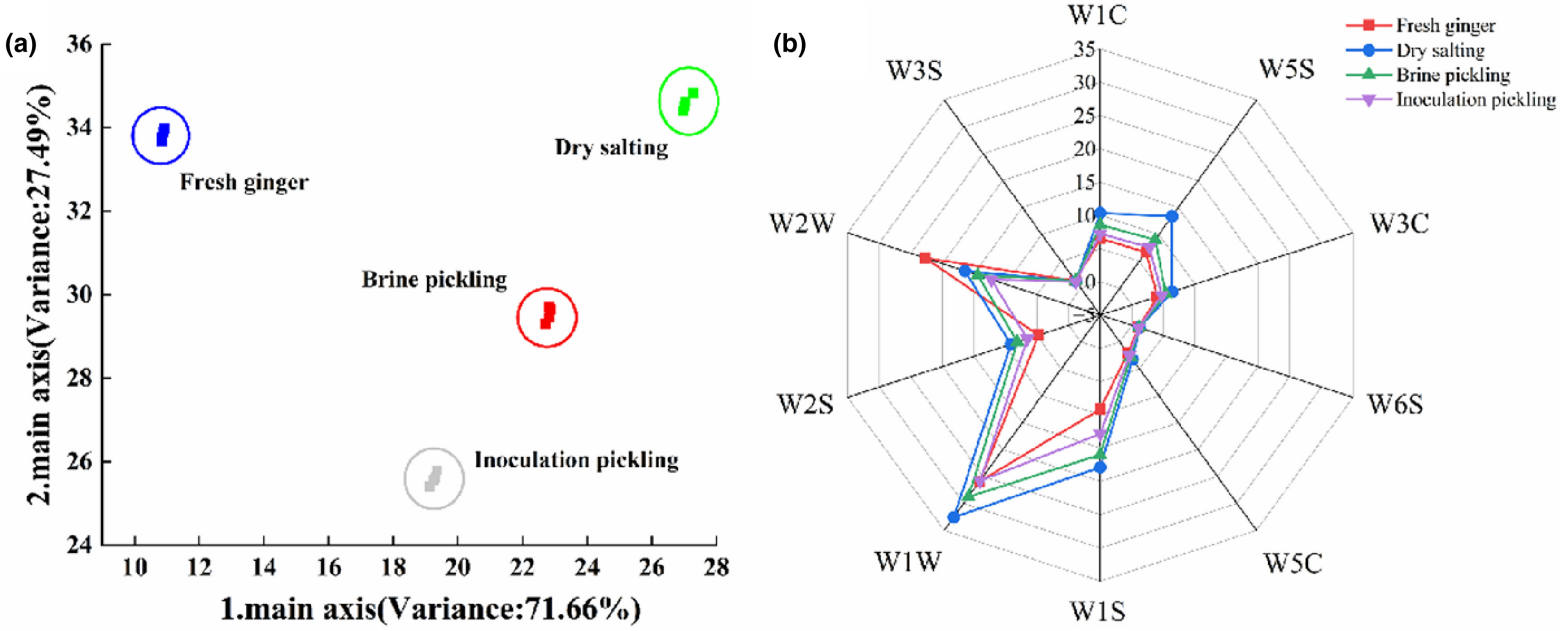
Principal component analysis (a) and radar chart (b) of the electronic nose data for Tongling white ginger at different fermentations.

Using an electronic nose, the characteristics of flavor in the samples were examined. The obtained results were subsequently subjected to PCA to provide a comprehensive summary of the relationship between attributes and samples. As depicted in Figure 3a, PC1 and PC2 contributed 71.66% and 27.49%, respectively. Based on PC1 and PC2's cumulative contribution rates, most information in the sample can be derived from them (Bai et al., 2021). The data points of the same sample in the figure were relatively well‐aggregated, indicating high repeatability and stability of the same sample. However, the sample area distribution was obviously different before and after fermentation, with significant separation between samples fermented using different methods. As a result, the volatile flavor components in the four samples could be properly and successfully distinguished using the electronic nose.

Subtle alterations in the volatile flavor compounds can result in variations in the responses measured by electronic olfactory sensors. As illustrated in Figure 3b, the response values of W1W, W1S, and W5S sensors exhibit significant differences. This indicates that the samples contain higher levels of terpenoids, sulfides, alkanes, and nitrogen oxides after fermentation. The results of volatile flavor determination showed that terpenoids also had the highest content. The response value of the W2W sensor also varies considerably, with fresh ginger exhibiting a higher response value than fermented white ginger. This suggests that the samples contain more organic sulfides and aromatic compounds. Contrarily, the W3S, W6S, and W5C sensors' response levels are modest, although the signal strength still differs. Therefore, electronic nose technology can effectively distinguish the volatile flavor of white ginger treated in different ways.

### 3.7 Volatile fraction

Food flavor is largely influenced by volatile flavor substances, which play a crucial role in people's preference and interest in food (Wang et al., 2022). Table 3 presents the results of 73 volatile compounds detected in dry‐salting, brine‐pickling, and inoculation‐pickling, but with varying contents. These compounds included 49 terpenoids, 10 esters, 7 alcohols, 3 aldehydes, 3 ketones, and 1 other compound.

**TABLE 3:** Volatile flavor compounds and their relative content during fermentation.

No.	Compounds	CAS	Content (μg/g)			
			Fresh ginger	Dry salting	Brine pickling	Inoculation pickling
Terpenoids						
1	δ‐Cadinene	483–76‐1	1721.72	1438.83	1559.15	1851.78
2	(Z)‐citral	106–26‐3	132.22	131.31	148.74	301.89
3	Epsilon‐muurolene	30021–46‐6	42.39	36.44	39.24	31.14
4	α‐Muurolene	10208–80‐7	1529.56	1328.74	1274.66	1584.92
5	Myrcenone	539–70‐8	5.38	5.98	–	6.31
6	D‐Carvone	2244‐16‐8	13.11	–	23.05	15.03
7	β‐Ocimene	13877–91‐3	2246.88	1771.88	1674.81	1746.19
8	1,8‐Cineole	470–82‐6	1040.59	817.84	838.3	871.85
9	Ascaridole	512–85‐6	62.47	15.01	134.15	79.55
10	Cubebol	23445–02‐5	813.24	677.34	694.34	864.74
11	β‐Phellandrene	555–10‐2	2244.7	1770.81	1673.73	1744.29
12	(+)‐Neodihydrocarveol	18675–33‐7	13.94	18.62	19.16	10.85
13	Eudesma‐2,4,11‐triene	82462–31‐5	421.48	351.37	396.44	764.28
14	δ‐Guaiene	3691‐11‐0	1030.49	882.47	974.25	881.64
15	(−)‐Trans‐beta‐bergamotene	15438–94‐5	1282.32	1098.03	1078.89	1330.66
16	(E)‐4,8‐dimethyl‐1,3,7‐nonatriene	19945–61‐0	114.12	86.95	63.8	63.18
17	β‐Bourbonene	5208‐59‐3	132.18	93.4	203.5	193.31
18	Bornyl acetate	76–49‐3	260.42	194.85	197.73	322.24
19	Citral	5392‐40‐5	1986.47	1581.03	1638.64	1395.86
20	α‐Zingiberene	495–60‐3	3253.27	2664.3	2704.67	3220.23
21	α‐Farnesene	502–61‐4	278.89	233.45	283.32	270.91
22	δ‐Elemene	20307–84‐0	47.74	40.56	34.86	31.41
23	Linalyl acetate	115–95‐7	144.91	37.8	303.42	182.8
24	Ledol	577–27‐5	89.14	68.21	71.71	75.14
25	Citrol	624–15‐7	1980.36	1581.79	1641.02	1397.05
26	(±)‐Menthone	89–80‐5	3.44	7.05	2.6	–
27	Terpinolene	586–62‐9	289.72	226.34	175.73	221.65
28	Bicyclogermacrene	24703–35‐3	667.11	579.56	555.99	695.86
29	Orthodene	4889‐83‐2	1846.84	1532.08	1379.88	1775.84
30	α‐Pinene	3856‐25‐5	633.42	492.68	367.6	479.39
31	(R)‐(+)‐beta‐citronellol	1117‐61‐9	30.22	38.88	41.14	23.05
32	α‐Pinene	80–56‐8	1821.07	1430.75	1188.7	1528.11
33	α‐Pyronene	514–94‐3	120.93	83.41	50.62	73.04
34	Neomenthoglycol	3564‐95‐2	55.05	58.92	113.6	92.87
35	α‐Curcumene	644–30‐4	1399.35	1170.67	1293.95	1784.44
36	(−)‐Isoledene	95910–36‐4	404.04	319.24	273.75	329.15
37	α‐Humulene	6753‐98‐6	227.77	185.27	164.61	217.56
38	10‐Epizonarene	41702–63‐0	736.63	636.42	701.23	767.01
39	β‐Guaiene	88–84‐6	1286.24	1054.51	1173.16	1122.38
40	Myrcene	123–35‐3	310.2	233.67	209.56	243.74
41	β‐Sesquiterpenes	20307–83‐9	1229.04	1032.52	1113.91	1367.85
42	Dihydromyrcene	2436‐90‐0	158.95	136	103.73	146.56
43	Sabinene	3387‐41‐5	759.64	546.62	350.58	487.13
44	β‐Pinene	127–91‐3	983.28	751.62	760.51	846.91
45	Pseudolimonene	499–97‐8	538.3	407.5	228.57	189.44
46	Beta‐cubebene	13744–15‐5	203.57	158.84	133.61	183.8
47	Dextro, laevo‐borneol	507–70‐0	588.01	550.6	382	602.58
48	cis‐α‐Bisabolene	29837–07‐8	1573.83	1346.01	1592.79	1545.98
49	Limonene	138–86‐3	522.93	453.05	346.05	551.4
Total concent (μg/g)			37,277.57	30,359.22	30,375.45	34,512.99
Ester						
50	(Z)‐3‐hexen‐1‐yl valerate	35852–46‐1	141.22	59.53	66.73	60.61
51	(E)‐benzyl tiglate	37526–88‐8	2132.16	2143.67	2539.39	2482.48
52	(Z)‐chrysanthenyl acetate	67999–48‐8	130.84	169.25	165.56	217.04
53	Geranyl isobutyrate	2345‐26‐8	915.85	1165.57	1041.24	1089.15
54	(1R,5S,7R)‐chrysanthenyl acetate	50764–55‐1	129.50	52.22	57.85	52.37
55	Benzyl formate	104–57‐4	115.33	115.80	90.18	148.66
56	Ethyl phenyl acetate	101–97‐3	18.01	52.26	76.36	49.99
57	Hexyl isovalerate	10032–13‐0	21.84	–	37.63	17.58
58	Octyl acetate	112–14‐1	5.94	–	2.91	–
59	Phenethyl acetate	103–45‐7	–	5.04	4.92	6.56
Total concent (μg/g)			3610.69	3763.34	4082.77	4124.44
Alcohol						
60	4‐Phenyl‐2‐butanol	2344‐70‐9	10.21	–	–	–
61	2‐Butyl‐2‐octenal	13019–16‐4	373.75	112.77	413.55	184.02
62	3‐Mercapto‐3‐methyl butanol	34300–94‐2	–	6.09	–	8.66
63	3‐Methyl catechol	488–17‐5	50.27	65.81	64.90	85.83
64	2,4‐Decadien‐1‐ol	14507–02‐9	652.44	745.09	791.32	946.34
65	Para‐cresyl alcohol	589–18‐4	14.14	19.92	12.47	22.71
66	Lilac pentanol	10415–87‐9	200.12	123.39	197.53	166.75
Total concent (μg/g)			1300.93	1073.07	1479.77	1414.31
Aldehyde						
67	Perillaldehyde	2111‐75‐3	71.09	87.75	88.94	112.19
68	(E)‐2‐dodecenal	20407–84‐5	14.68	18.26	19.61	21.45
69	(Z)‐2‐decenal	2497‐25‐8	240.68	47.78	406.22	195
Total concent (μg/g)			326.45	153.79	514.77	328.64
Ketone						
70	(E)‐filbertone	102322–83‐8	86.76	97.54	62.27	140.39
71	Para‐ethyl acetophenone	937–30‐4	–	6.60	6.09	8.71
72	2‐Cyclopentyl cyclopentanone	4884‐24‐6	687.47	840.13	854.73	1077.54
Total concent (μg/g)			774.23	944.27	923.09	1226.64
Others						
73	5‐(Methyl thio) valeronitrile	59,121–25‐4	16.14	–	7.50	–
Total concent			16.14	–	7.50	–

*Note*: –, could not be detected.

**FIGURE 4:**
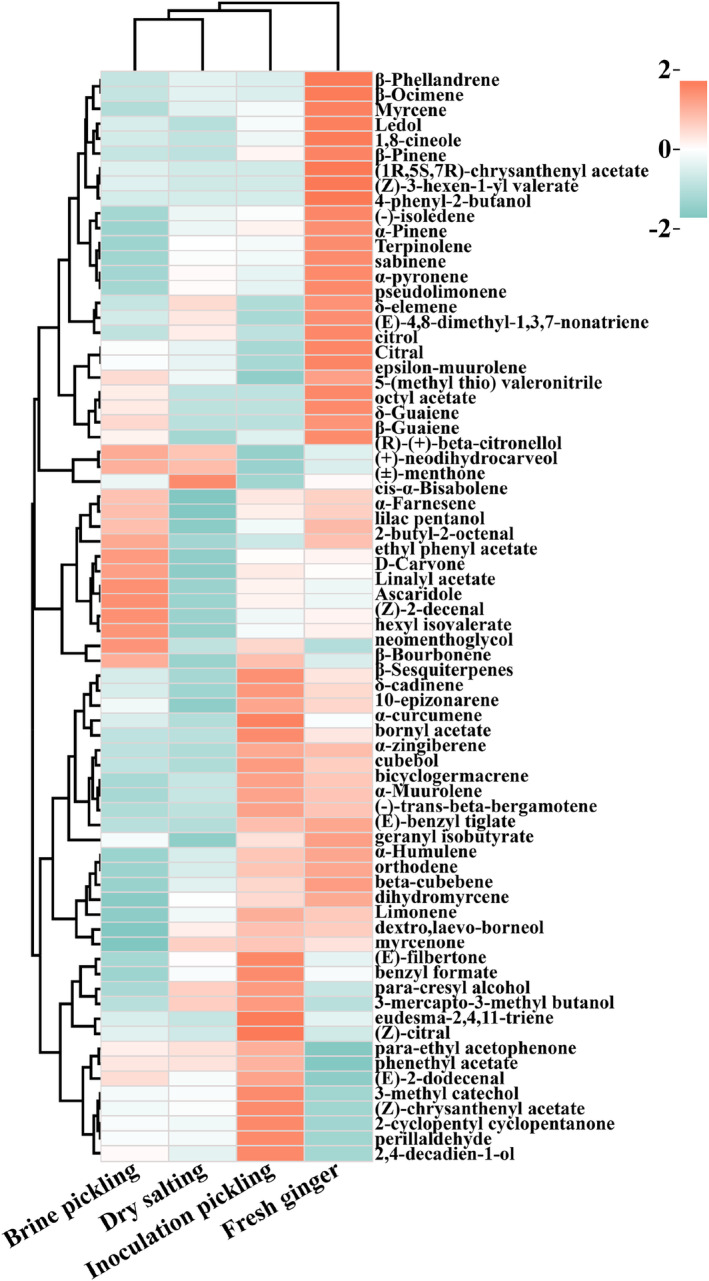
Cluster heat map of volatile flavor substances of Tongling white ginger by different fermentations.

The heat map was sketched using ChiPlot software (Figure 4). The overall aroma composition could be analyzed after content data were standardized and plotted. When the range of the ingredient content was low to high, there was a color of red to blue gradient in a heat map. The compositional abundance distribution of the samples could be visualized in Figure 4, where the dry‐salting and brine‐pickling were grouped together on the same branch, indicating that their volatile components were similar (Padilla‐Jiménez et al., 2021). The fresh ginger was not grouped in the same branch as any fermented white ginger, indicating that the distribution of volatile components in fresh ginger differed from several fermented white gingers. Inoculation‐pickling was closer to fresh ginger and dry‐salting, but it was not in the same branch. It indicates that inoculation‐pickling produces a distinctive flavor, without losing the flavor of the white ginger itself.

The total amount of different types of volatile components in the four types of samples (fresh ginger, dry‐salting, brine‐pickling, and inoculation‐pickling) was dominated by terpenoids, esters, and alcohols. Of the fresh ginger, dry‐salting, brine‐pickling, and inoculation‐pickling, *α*‐zingiberene was also the highest (3253.27, 2664.30, 2704.67, and 3220.23 ug/g, Table 3), which was in line with the findings of research by Luo et al. (2017) on the volatile components of ginger. *α*‐Zingiberene is mainly spicy and has a fresh flavor, and the characteristic aroma of white ginger itself, and thus the highest content in fresh ginger. The content decreases after fermentation, indicating a reduction in spiciness. The four samples detect many advanced aromatic terpenes, for example, *β*‐phellandrene, α‐farnesene, α‐pinene, and α‐curcumene, with a pleasant, distinctive scent of ginger. (±)‐Menthone was presented at a maximum of 7.05 μg/g in the dry‐salting, mainly presenting a menthol flavor. Brine‐pickling was more prominent in D‐carvone, *β*‐bourbonene, linalyl acetate, and (R)‐(+)‐beta‐citronellol, which presented mint, floral, lavender, and rose fragrances, respectively. The *δ*‐cadinene and *β*‐sesquiterpene contents from inoculation‐pickling were the highest and gave thyme and citrus aromas, respectively.

They have the second‐highest ester content, and they were found to be the key contributors to the distinctive flavor of pickled vegetables (Wu et al., 2015). The high content of esters in ginger is the main contributor to its odor. The four samples (fresh, dry‐salting, brine‐pickling, and inoculation‐pickling) contained the highest levels of benzyl tailgate (2132.16, 2132.16, 2539.39, and 2482.48 μg/g, respectively; Table 3). It is also the second only to α‐zingiberene in all flavor compounds, with mushroom and nut flavors. The total number of esters increased after fermentation, probably due to the pickling process leading to the formation of some esters through esterification reactions in the presence of microorganisms (Chen, Luo, Niu, et al., 2019; Chen, Luo, Peng, et al., 2019). Noteworthy, although the total amount was reduced, phenethyl acetate as a new compound appeared after fermentation, presenting a rose scent.

The alcohols in the four samples were mainly 2,4‐decadien‐1‐o and 2‐butyl‐2‐octenal, adding a sweet, honeyed flavor to white ginger. The alcohols in the four samples were mainly 2,4‐decadien‐1‐o with a fatty aroma. Alcohol content decreased with dry‐salting and increased in brine‐pickling and inoculation‐pickling. There was the highest total content in brine‐pickling (1479.77 ug/g). This was due to the high number of miscellaneous bacteria and the slow start of fermentation in brine‐pickling fermentation. Alcohol accumulation is too much, which is easy to grow for yeast and other miscellaneous bacteria as a result of the easy production of alcohol. Compared to brine‐pickled, fermentation of inoculation‐pickling started fast, resulting in fast alcohol consumption. The dominant bacteria inhibited the growth of yeast and other miscellaneous bacteria, so the alcohol content of the inoculation‐pickling white ginger was low. The highest total aldehyde and ketone flavor compounds were found in brine‐pickling and inoculation‐pickling. Probably lactic acid bacteria's active metabolism during fermentation may impair other types of microorganisms' growth and reproduction, resulting in the production of aldehydes and ketones as intermediate products.

### 3.8 Sensory evaluation

**FIGURE 5:**
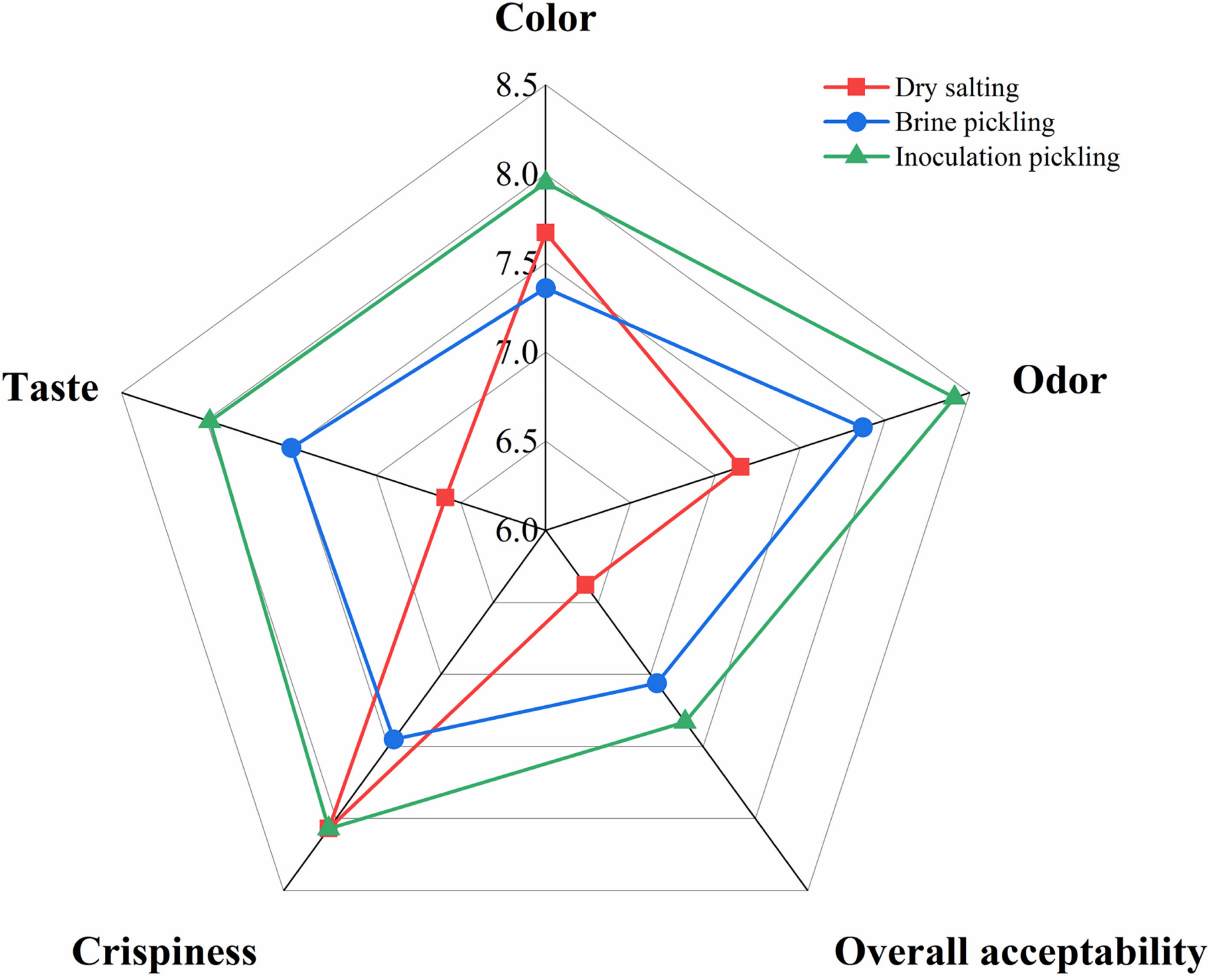
Sensory scores of different fermented Tongling white ginger.

A radar diagram (Figure 5) represents the results of sensory evaluation. Dry‐salting compared with brine‐pickling: dry‐salting scored higher for color (7.67) and crispness (8.07), while brine‐pickling scored higher for odor (7.87) and texture (7.50). However, inoculation‐pickling had the highest color, odor, and taste scores of 7.95, 8.41, and 7.98, respectively. The fermented aroma of white ginger was found in both brine‐pickling and inoculation‐pickling, the dry‐salting aroma was not prominent, but none of any other off‐flavors. In terms of overall acceptability, the two brine pickles had higher sensory scores than the dry salt, with the inoculation‐pickled being the highest at 7.33. In summary, inoculation‐pickling had the highest sensory scores. Their volatile scent components' kind and concentration may have an impact on this outcome.

## 4 CONCLUSION

The effects of pickling with brine, inoculation, and dry salting on the physical and chemical properties, non‐volatile qualities, and flavor attributes of white ginger vary. During the three salting processes, the TA value significantly increased while pH significantly decreased. Additionally, the color changed significantly, and the nitrite content declined after the “nitrite peak”, falling to safe consumption standards. Inoculation pickling was more beneficial than dry salting and brine pickling in accelerating vegetable fermentation and shortening the maturity period. As a result of this process, reducing sugars turn them into organic acids, resulting in the richness of lactic acid and a unique sour taste in the inoculation‐pickled white ginger. White ginger pickled in dry salt had a lower water content than brine‐pickled and inoculation‐pickled ginger, resulting in a higher level of non‐volatile amino acids. Inoculation pickling contributes to the formation of non‐volatile substances, and volatile flavor contents were the highest. In the volatile heat map cluster analysis, dry salting and brine pickling were grouped into the same branch. Inoculation pickling produces a distinctive flavor, without losing the flavor of the white ginger itself. The electronic nose can also distinguish the volatile flavor of fresh ginger from different pickled white ginger effectively and is more consistent with the volatile flavor results. In the sensory score, it was concluded that the sensory score of inoculation‐pickled white ginger was better, and the overall acceptable score was the highest. This study revealed the physicochemical characteristics, non‐volatile flavor, and volatile flavor characteristics of dry salting, brine pickling, and inoculation pickling, which provides a basis for further understanding the effects of the three salting processes.

## AUTHOR CONTRIBUTIONS

**Kaili Zong:** Conceptualization (equal); data curation (equal); formal analysis (equal); funding acquisition (equal); investigation (equal); methodology (equal); software (equal); validation (equal); writing – original draft (equal); writing – review and editing (equal). **Feixiang Jin:** Formal analysis (equal); investigation (equal); methodology (equal). **Daquan Wang:** Investigation (equal); methodology (equal); validation (equal); writing – review and editing (equal). **Hongchao Hu:** Investigation (equal); methodology (equal); writing – review and editing (equal). **Haipeng Cui:** Investigation (equal); methodology (equal); writing – review and editing (equal). **Jianting Yang:** Conceptualization (equal); funding acquisition (equal); project administration (equal); resources (equal); supervision (equal); writing – review and editing (equal).

## CONFLICT OF INTEREST STATEMENT

The authors have no conflict of interest to declare.

## PRACTICAL APPLICATION

The findings of this study will contribute to a better understanding of the effects of brine pickling, dry salting, and inoculation pickling on the quality and flavor of Tongling white ginger.

## Data Availability

The data that support the findings of this study are available on request from the corresponding author. The data are not publicly available due to privacy or ethical restrictions.
